# Supplementary material for: Induction of synapse formation by de novo neurotransmitter synthesis
Source: Nat Commun. 2022 Jun 1;13:3060. doi: 10.1038/s41467-022-30756-z (PMC9160008; doi:10.1038/s41467-022-30756-z)
Supplement: Supplementary file 1 — Supplementary Information [file 41467_2022_30756_MOESM1_ESM.pdf]

Supplementary Figures & Figure Legends

Induction of Synapse Formation by *De Novo* Neurotransmitter Synthesis

Scott R. Burlingham[1,*], Nicole F. Wong[2,*], Lindsay Peterkin[1,*], Lily Lubow[1], Carolina Dos Santos Passos[1], Orion Benner[1], Michael Ghebrial[3], Thomas P. Cast[1], Matthew A. Xu-Friedman[2,#], Thomas C. Südhof[4,#], and Soham Chanda[1,4,5,#,$]

[1]Biochemistry & Molecular Biology, Colorado State University, Fort Collins, CO 80523, USA;
[2]Biological Sciences, State University of New York at Buffalo, Buffalo, New York 14260, USA;
[3]Biological Science, California State University Fullerton, Fullerton, CA 92831, USA;
[4]Molecular and Cellular Physiology, Stanford University School of Medicine, Stanford, CA 94305, USA;
[5]Molecular, Cellular & Integrated Neurosciences, Colorado State University, Fort Collins, CO 80523, USA;

*These authors contributed equally: SRB, NFW, LP
[#]Correspondence: SC (soham.chanda@colostate.edu); TCS (tcs1@stanford.edu); MAX (mx@buffalo.edu)
[$]Lead Contact: SC (soham.chanda@colostate.edu)

List of Supplementary Figures:

Supplementary Figure S1 (Related to Figure 1 and Figure 2)**:**
Glutamatergic human neurons lack presynaptic enzymes for GABA release.

Supplementary Figure S2 (Related to Figure 1 and Figure 2)**:**
V57 factors alter the identity of synaptic currents in glutamatergic neurons.

Supplementary Figure S3 (Related to all Figures)**:**
Primary antibody information.

Supplementary Figure S4 (Related to Figure 3)**:**
V57 factors do not affect neuronal maturation or morphology.

Supplementary Figure S5 (Related to Figure 3 and Figure 4)**:**
Glutamatergic vs. GABAergic synapses exhibit minimal co-localization.

Supplementary Figure S6 (Related to Figure 3 and Figure 4)**:**
V57 factors promote alignment of GABAergic synapse components.

Supplementary Figure S7 (Related to Figure 5)**:**
Prolonged PTX exposure impairs GABAergic synapse morphology.

Supplementary Figure S8 (Related to Figure 6)**:**
GABAergic synapse formation by V57 factors in iPS cell -derived neurons.

Supplementary Figure S9 (Related to Figure 7)**:**
In vivo virus injection into mouse spiral ganglion neurons.

Supplementary Figure S10 (Related to all Figures)**:**
Schematic diagram of direct conversion of synapse identity by V57 factors.

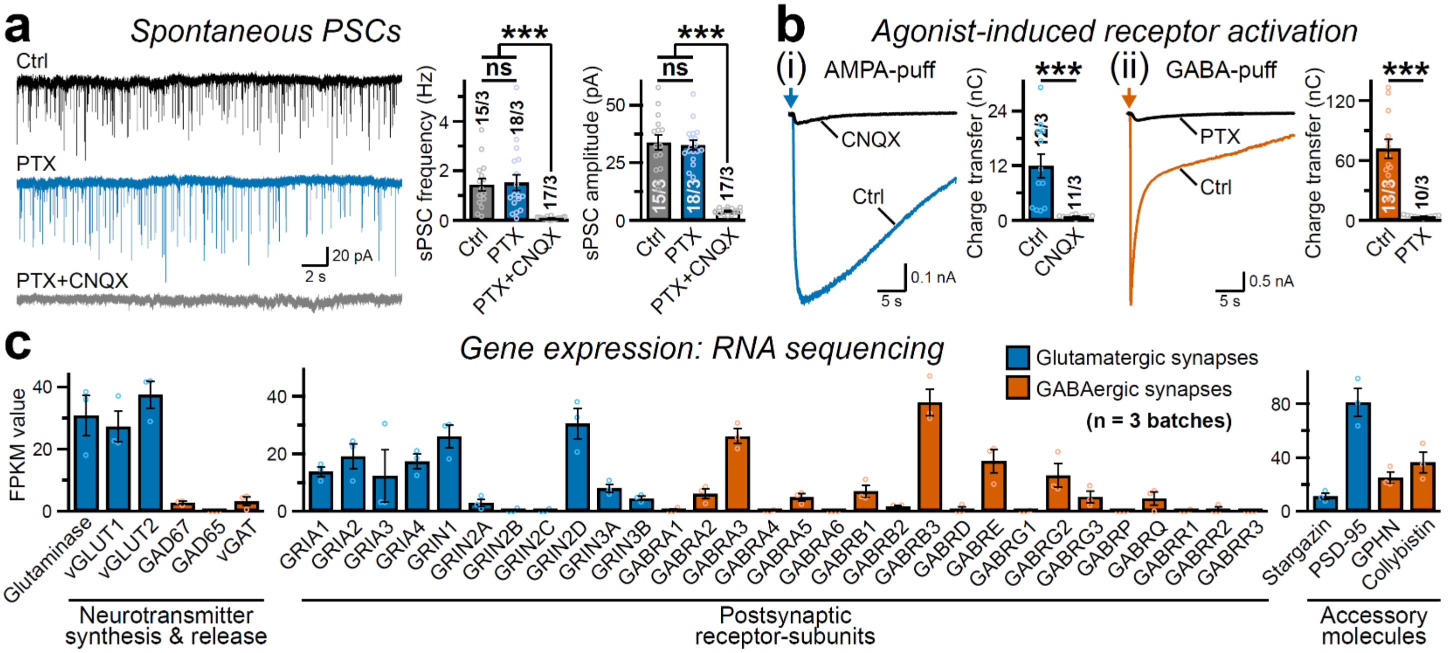

Supplementary Figure S1: **Glutamatergic human neurons lack presynaptic enzymes for GABA release.**

Pure glutamatergic neurons were obtained from direct reprogramming of human H1-ES cells by forced expression of a single transcription factor Ngn2[1], and analyzed at post-induction day 56-60 (see Fig. 1a).

a. Representative traces (left), average frequency and amplitude (right) of sPSC events recorded from Ngn2-induced human neurons at post-induction day 56-60, before (Ctrl) or after acute treatments of PTX and CNQX.

b. Sample traces (left) and average charge-transfer (right) of currents produced by exogenous applications of AMPA (**i**) or GABA (**ii**), each puffed at 1 mM, that were subsequently blocked by CNQX or PTX, respectively.

c. FPKM values of pre- and postsynaptic genes associated with either glutamatergic or GABAergic synapses, as curated from our previous RNA-sequencing experiment on Ngn2-only neurons at post-induction day 56-60[2].

All quantifications represent means ± SEM; open circles on the bar-graphs indicate individual data-points, with number of neurons patched / independent batches (**a**, **b**), or only the number of batches (for **c**). Skewness and Kurtosis (-2 >≈ values ≈< 2) were consistent with near-normal distribution (Source Data); statistical significance was assessed by two-tailed, unpaired, Student's t-test (*** $P < 0.005$; ns = not significant, $P > 0.05$).

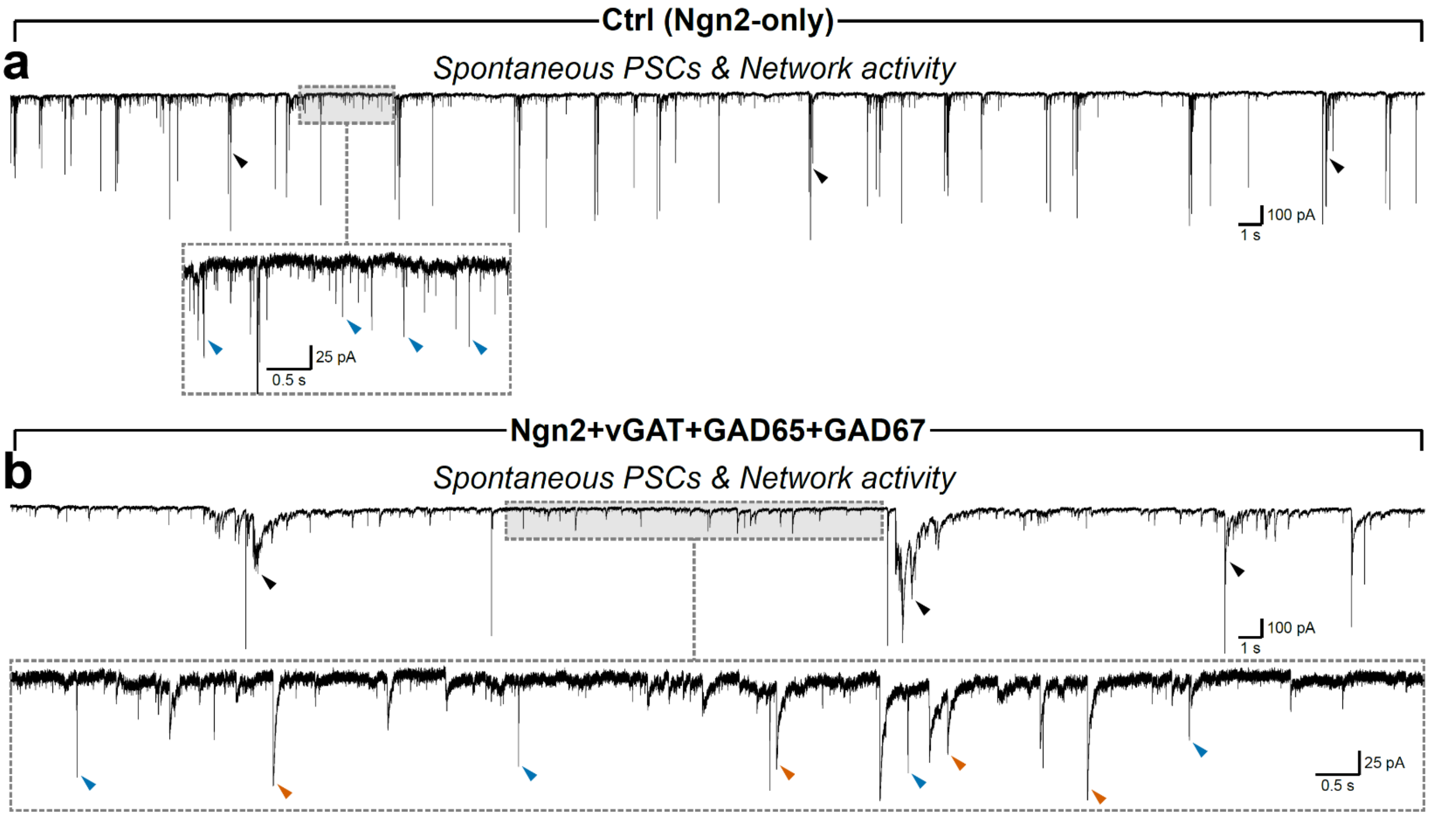

Supplementary Figure S2: **V57 factors alter the identity of synaptic currents in glutamatergic neurons.**

a. Example trace of sPSC recordings from day 56-60 human neurons expressing Ngn2-only.

b. Same as **a**, except for Ngn2-induced glutamatergic neurons co-transduced with V57 factors.

Black arrowheads point at bursts of sPSCs generated by recurrent network activities, usually produced due to spontaneous AP-firing in neighboring neurons; Insets = boxed regions expanded, with blue vs. red arrowheads indicating sPSC events respectively with fast vs. slow τ-decays, independent of the network activities.

a *List of primary antibodies*

Antibody	Species	Vendor	Catalog #	Dilution
Calretinin	Goat	Swant	CG1 (Lot # 1§.1)	1:1000
EGFP	Chicken	Aves Labs	GFP-1020	1:1000
GABRA3	Rabbit	Abclonal	A11636	1:500
GAD65	Rabbit	Abclonal	A0971	1:500
GAD67	Rabbit	Abclonal	A2938	1:500
Gephyrin [#1]	Mouse	Synaptic Systems	147011 (Clone: mAb7a)	1:500
Gephyrin [#2]	Mouse	Synaptic Systems	147111 (Clone: 3B11)	1:500
Homer-1 [#1]	Mouse	Synaptic Systems	160011 (Clone: 2G8)	1:500
Homer-1 [#2]	Rabbit	Synaptic Systems	160002	1:500
HuNu	Mouse	Millipore Sigma	MAB1281 (Clone: 235-1)	1:500
MAP2	Chicken	Abcam	Ab5392	1:1000
Neuroligin-2	Rabbit	Synaptic Systems	129203	1:500
RFP	Rabbit	Rockland	600-401-379	1:500
Synapsin-1 [#1]	Mouse	Synaptic Systems	106011 (Clone: 46.1)	1:1000
Synapsin-1/2 [#2]	Guinea Pig	Synaptic Systems	106004	1:500
Synapsin-1/2 [#3]	Rabbit	Synaptic Systems	106002	1:500
Tuj1 (βIII-tubulin)	Mouse	BioLegend	801202 (Clone: TUJ1)	1:400/1000
vGLUT1	Rabbit	Synaptic Systems	135303	1:500
vGAT [#1]	Mouse	Synaptic Systems	131011 (Clone: 117G4)	1:500
vGAT [#2]	Rabbit	Synaptic Systems	131003	1:500

b *Antibody specificity in human neurons*

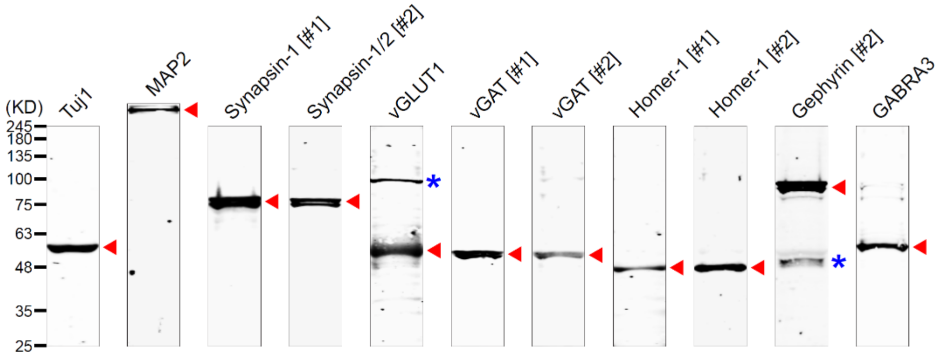

Supplementary Figure S3: **Primary antibody information.**

a. List of all primary antibodies used at their corresponding dilutions. For commercially available monoclonal antibodies, the clone/lot #s (in parenthesis) are provided along with their vendor information and catalog #s.

b. Whole-cell lysates of Ngn2-neurons co-expressing V57 factors (day 56-60) were probed with respective antibodies. Red arrowheads = expected molecular weights of proteins, blue asterisks = nonspecific products. All band patterns were successfully reproduced in 2-4 batches of cell lysates extracted from NV57 neurons.

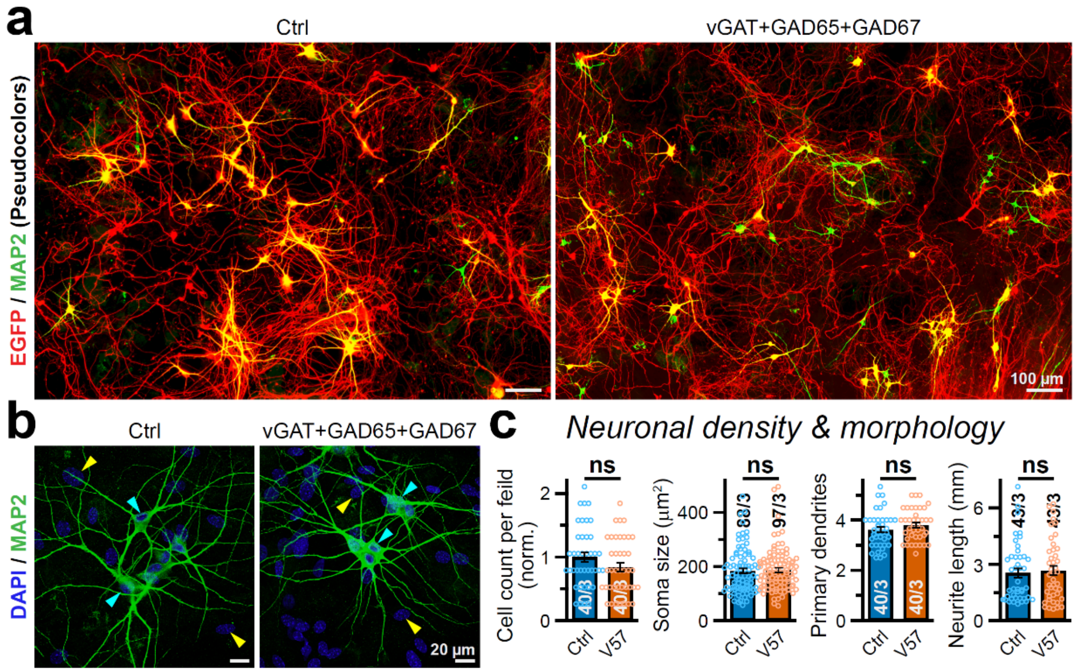

Supplementary Figure S4: **V57 factors do not affect neuronal maturation or morphology.**

a-b. Whole-field views (**a**) and magnified images (**b**) of Ngn2-only (Ctrl) vs. NV57 neurons labeled with EGFP expression, co-stained for MAP2 and DAPI. In **b**, cyan arrowheads = neurons, yellow arrowheads = glial cells.

c. Average values (left to right panels) of normalized cell-density (left), soma-size (middle left), number of primary dendrites (middle right), and total dendritic length (right) per cell, for Ngn2-only vs. NV57 conditions.

All average values indicate mean ± SEM, with total number of field-of-views analyzed / number of independent experimental batches, and individual data-points included as color-coded open circles. Skewness and Kurtosis values (-2 >≈ and ≈< 2) suggested an approximately normal distribution of the data-points (see Source Data). Statistical significance was calculated by two-tailed, unpaired, Student's t-test (ns = not significant, $P > 0.05$).

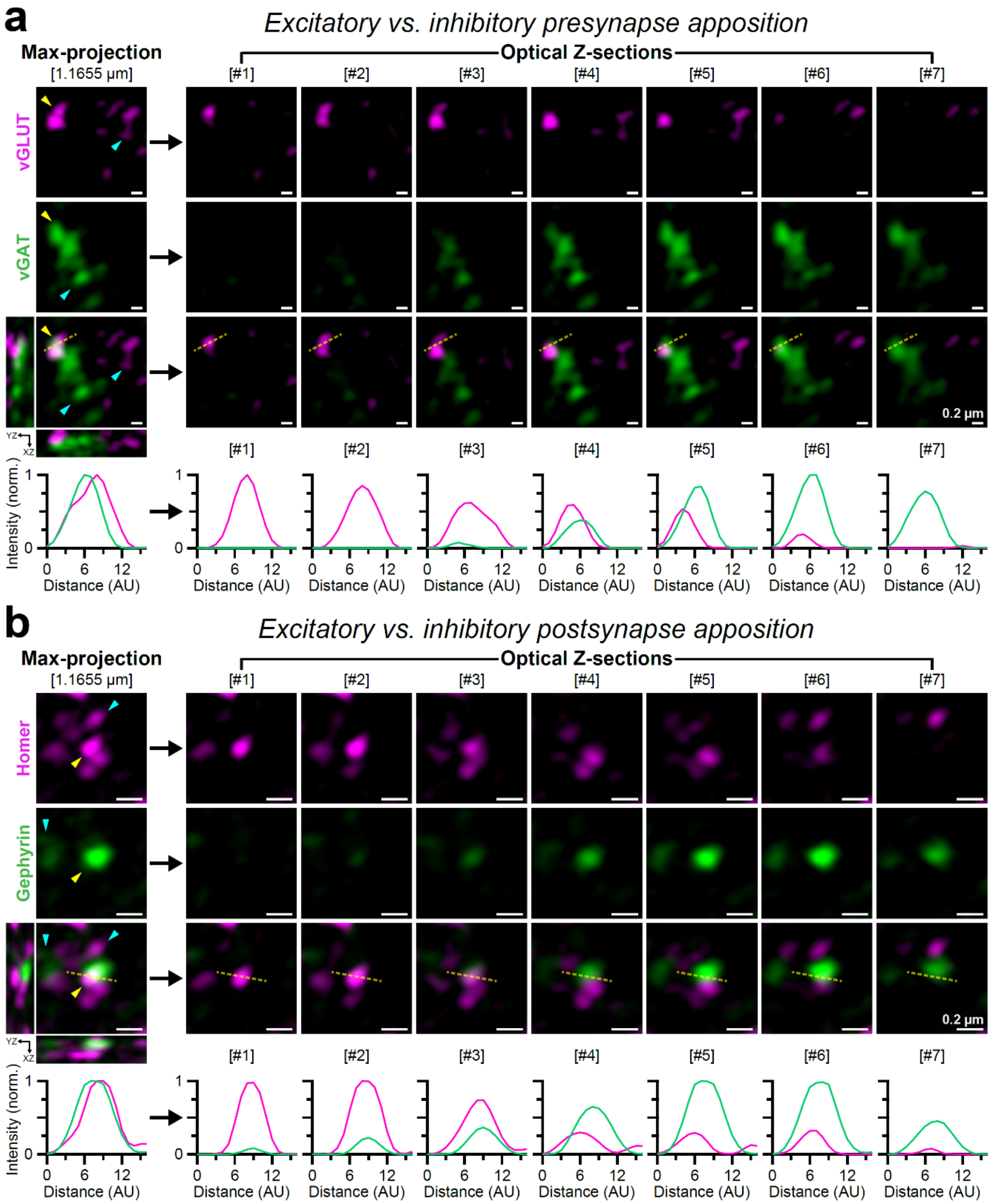

Supplementary Figure S5: **Glutamatergic vs. GABAergic synapses exhibit minimal co-localization.**

All images were acquired from Ngn2-induced glutamatergic human neurons that were additionally transduced with V57 factors. Images were processed using a Zeiss super-resolution microscope with Airyscan detector. The marker distribution patterns in panels **a** and **b** were reproducible in 3 batches, as quantified in Fig. 3f, j.

a. Maximum intensity z-projection (left) and individual optical sections (#1-7, right) display presynaptic puncta, glutamatergic vGLUT and GABAergic vGAT, that mostly occupy different synaptic zones (cyan arrowheads). Although these two signals occasionally appeared to co-localize on x/y focal plane (yellow arrowheads, max-projection), they could be further resolved as separate entities on x/z or y/z dimension (left and bottom insets), as they appeared distinctly at different z-planes (normalized intensity profile, dotted line, AU = arbitrary unit).

b. Same as **a**, except for postsynaptic markers, i.e. glutamatergic Homer and GABAergic Gephyrin.

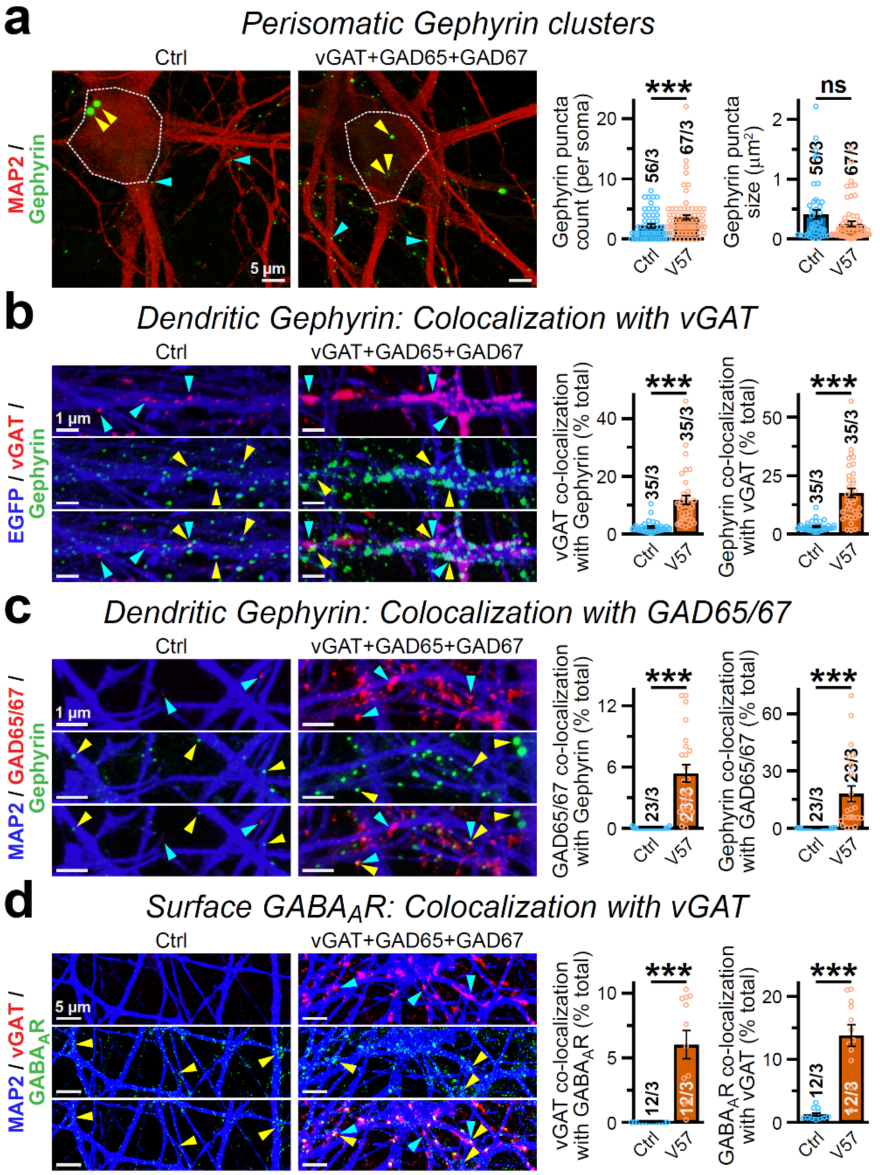

Supplementary Figure S6: **V57 factors promote alignment of GABAergic synapse components.**

a. Sample images (left) or average parameters (right) of Gephyrin clusters (yellow arrowheads) located in the perisomatic region (dotted area) of Ngn2-only control vs. NV57 neurons. Cyan arrowheads, dendritic Gephyrin.

b. Representative images (left) and Mander's coefficients (right) of co-localization between Gephyrin and vGAT signals along EGFP-labeled neurites (pseudo-colored), as monitored from Ngn2-only (Ctrl) vs. NV57 condition.

c. Same as **b**, except for co-localization between Gephyrin and GAD65/67, along MAP2-positive dendrites.

d. Same as **b**, except for co-localization between vGAT and surface GABA$_A$R, on MAP2-positive dendrites.

Averages on summary graphs represent mean ± SEM; the numbers on bar-graphs denote total field-of-views analyzed / number of experimental batches, individual data-points are provided as color-matched open circles. Statistical significances were measured using two-tailed, unpaired, nonparametric Mann-Whitney U-test (for all non-normal data distributions, see Source Data), with *** $P < 0.005$; ns = not significant, $P > 0.05$.

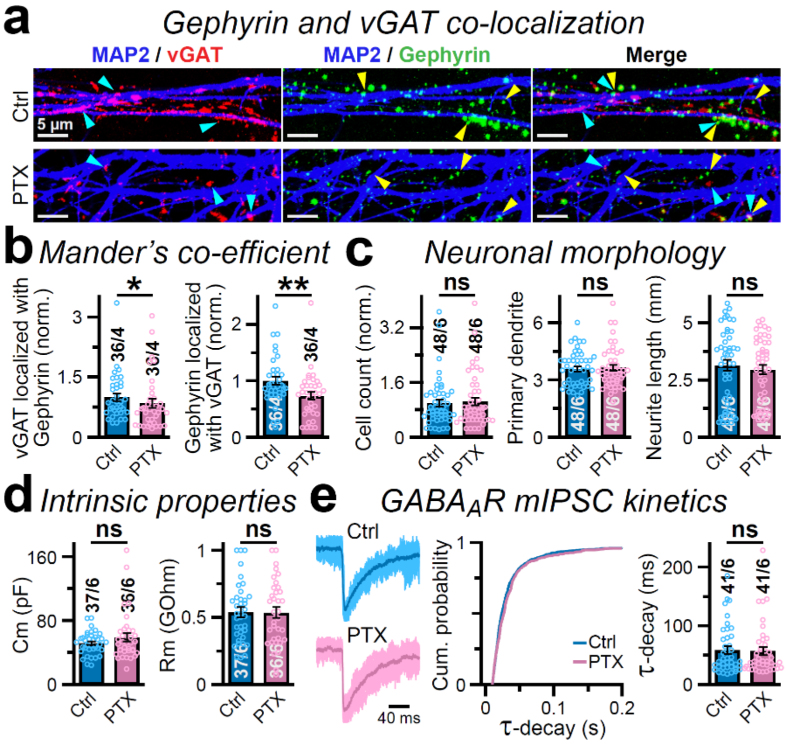

Supplementary Figure S7: **Prolonged PTX exposure impairs GABAergic synapse morphology.**

Glutamatergic human neurons were generated from H1-ES cells by Ngn2 transgene, co-transduced with V57 factors, incubated with PTX from post-induction day 4-5 to day 56-60, and examined afterwards (see Fig. 5a).

a-b. Representative images (**a**) and Mander's coefficients (**b**) of co-localization between vGAT (cyan arrowheads) and Gephyrin (yellow arrowheads) signals, without (Ctrl) or with chronic PTX incubation.

c. Summary graphs represent normalized density of neurons per field-of-view (left), number of primary dendrites (middle), and total dendritic length (right) per neuron, for control vs. PTX-treated conditions.

d. Average values of C_m (left) and R_m (right), for control vs. PTX-treated conditions.

e. Example mIPSC events (10 traces, light shades) with superimposed average (dark shades) waveform (left), cumulative distribution (middle) and summary graphs (right) of mIPSC τ-decay, for control vs. PTX-treatment.

All quantifications are means ± SEM. Numbers inside the bar-graphs indicate either field-of-views analyzed (for imaging) or cells patched (for electrophysiology) / number of independent batches. Corresponding data-points are plotted as open circles. Statistical significance was assessed using two-tailed, unpaired, Student's t-test, or two-sided, Mann-Whitney U-test, with * $P < 0.05$; ** $P < 0.01$; ns = not significant, $P > 0.05$ (see Source Data).

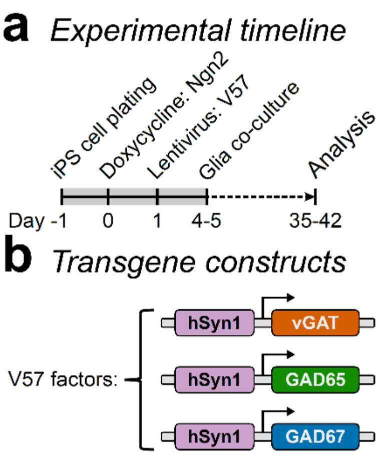

c qRT-PCR primer sequence

Genes	Forward Primer seq.	Reverse Primer seq.	Size
GAPDH	TTGAGGTCAATGAAGGGGTC	GAAGGTGAAGGTCGGAGTCA	117 bp
vGAT	CGACAAGCCCAAAATCACGG	GCCGTCTTCATTCTCCTCGT	199 bp
GAD65	TTCCTTTCCTCGTGAGTGCC	TCTCCACGCCACTCAGTTTC	174 bp
GAD67	AATACCACTAACCTGCGCCC	CTTCCAGGCTGTTGGTCCTT	118 bp

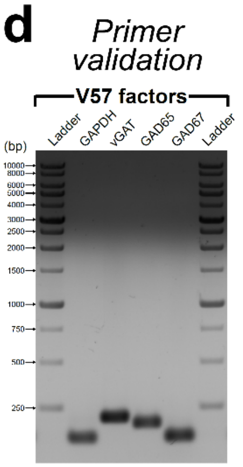

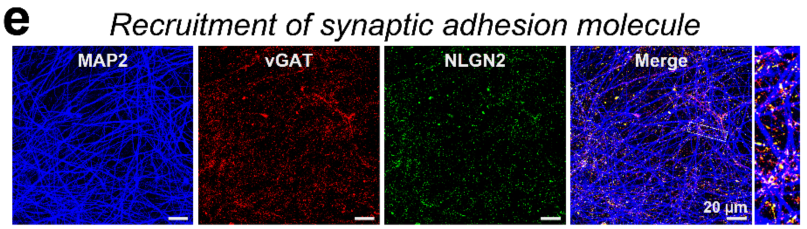

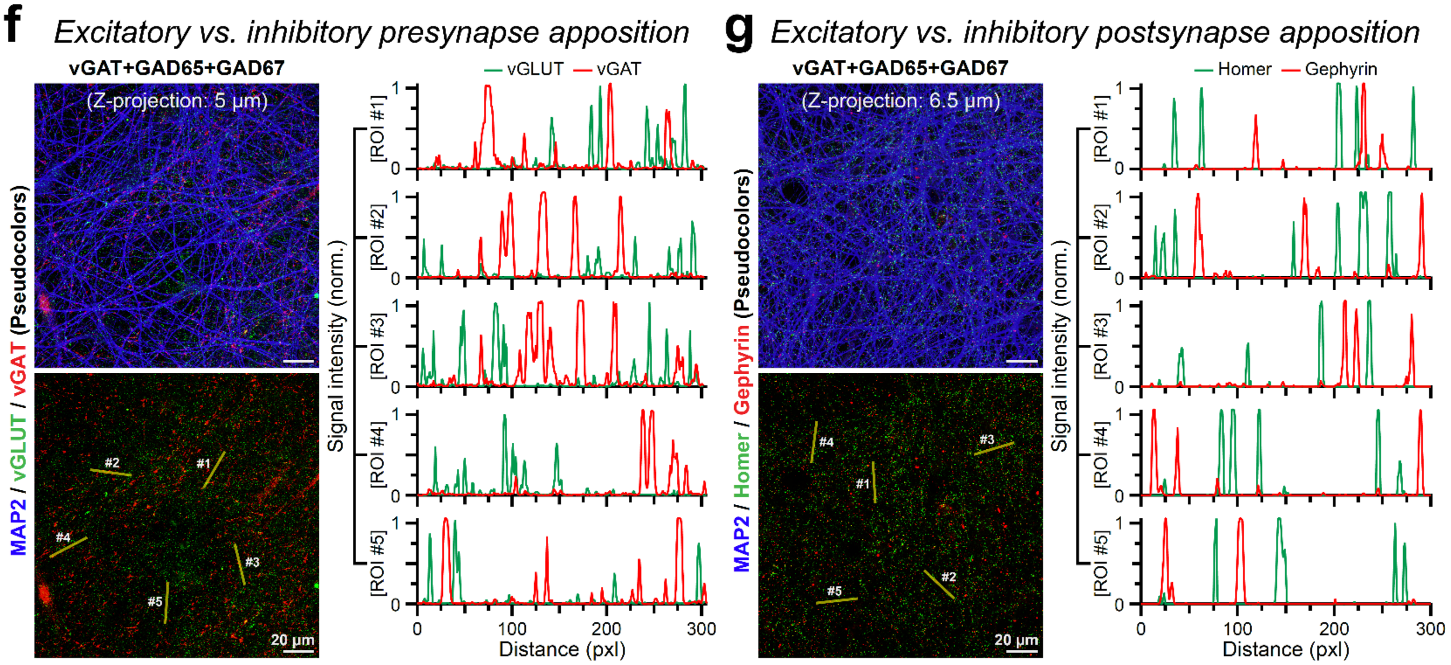

Supplementary Figure S8: **GABAergic synapse formation by V57 factors in iPS cell -derived neurons.**

a-b. Experimental protocol (**a**) and transgene construct designs (**b**) to express V57 factors in iPS cell (WTC-11 line) -derived Ngn2-neurons. They were co-cultured with glia and evaluated at post-differentiation day 35-42.

c-d. Sequence information of the primers with predicted size (**c**) and validation of qRT-PCR products, when run on an 1% agarose gel (**d**) for vGAT, GAD65, GAD67 transgenes, and a normalization control GAPDH. These band patterns were consistent for all 3 independent batches of cells prepared and analyzed in Fig. 6b.

e. iPS cell -derived human neurons transduced with V57 factors were co-immunostained for indicated markers; presynaptic vGAT signals showed a high degree of co-localization with Neuroligin-2 (NLGN2), a GABAergic postsynaptic SAM. This experiment was repeated in 4 independent biological replicates, with similar results.

f-g. Sample images (left) and normalized intensity profiles (right) of regions-of-interest (yellow lines, numbered) from neurons transduced with V57 factors, as immunolabeled for presynaptic (vGLUT in green, vGAT in red; **f**) or postsynaptic (Homer in green, Gephyrin in red; **g**) markers for glutamatergic vs. GABAergic synapses. Both marker pairs of different synaptic identities manifest little co-appearance, even in z-projected wide-field images. These cultures were also analyzed using super-resolution microscopy (3 batches, Fig. 6d), with similar results.

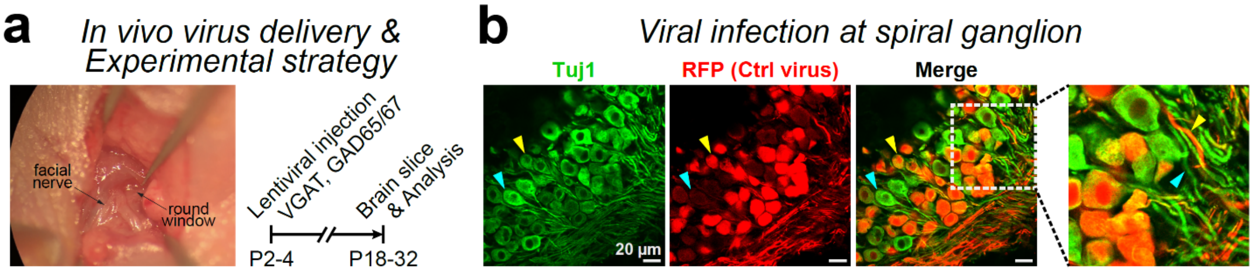

Supplementary Figure S9: ***In vivo* virus injection into mouse spiral ganglion neurons.**

a. Viral injection into the round window of neonatal mice (left); brain slices were studied after 2-4 weeks (right).

b. A control virus encoding RFP demonstrated successful but partial infection at the injection site, that was co-immunostained with Tuj1 antibody. Arrowheads point at infected (yellow) vs. uninfected (cyan) spiral ganglion neurons, and AN fibers originating from them (magnified). These AN fibers (VIIIth nerve bundles) subsequently project to the cochlear nuclei and form presynaptic endbulb terminals on postsynaptic BCs (see Fig. 7a). Viral injection was repeated in 6 animals (3 mice each for control vs. V57), and efficiency was assessed (Fig. 7b, c).

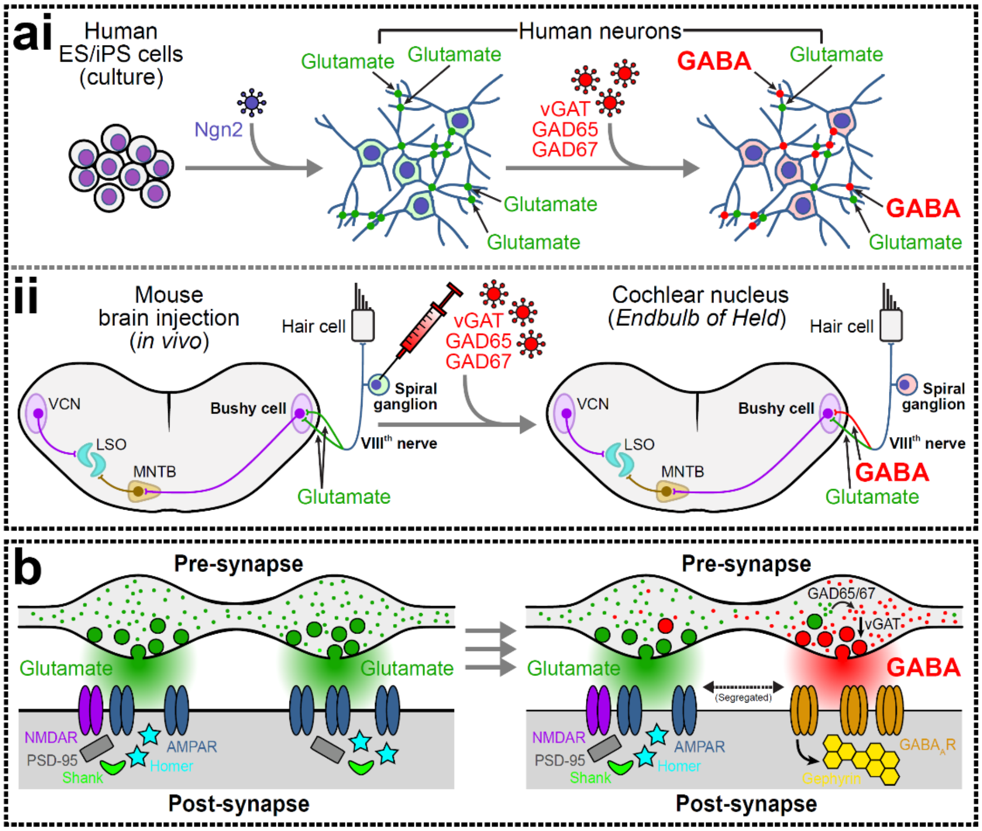

Supplementary Figure S10: **Schematic diagram of direct conversion of synapse identity by V57 factors.**

a. Glutamatergic human neurons reprogrammed from stem cells (**i**) or mouse neurons located in the cochlear spiral ganglion (**ii**) were acutely modified to produce GABAergic output synapse by viral delivery of V57 factors.

b. Model: Ectopic expression of GABA-synthesis enzymes and vesicular transporters can efficiently synthesize GABA from available glutamate and release it from presynaptic terminals. This triggers $GABA_AR$ activation and assembly of GABAergic postsynaptic apparatus, that organizes independently from glutamatergic synapses.

SUPPLEMENTARY REFERENCES

1 Zhang, Y. *et al.* Rapid single-step induction of functional neurons from human pluripotent stem cells. *Neuron* **78**, 785-798, doi:10.1016/j.neuron.2013.05.029 (2013).

2 Chanda, S. *et al.* Direct Reprogramming of Human Neurons Identifies MARCKSL1 as a Pathogenic Mediator of Valproic Acid-Induced Teratogenicity. *Cell Stem Cell* **25**, 103-119.e106, doi:10.1016/j.stem.2019.04.021 (2019).
