# Supplementary material for: Induction of synapse formation by de novo neurotransmitter synthesis
Source: Nat Commun. 2022 Jun 1;13:3060. doi: 10.1038/s41467-022-30756-z (PMC9160008; doi:10.1038/s41467-022-30756-z)
Supplement: Supplementary file 3 — Reporting Summary [file 41467_2022_30756_MOESM3_ESM.pdf]

nature portfolio

Corresponding author(s): Soham Chanda, Thomas C. Südhof, Matthew A. Xu-Friedman

Last updated by author(s): May 1, 2022

Reporting Summary

Nature Portfolio wishes to improve the reproducibility of the work that we publish. This form provides structure for consistency and transparency in reporting. For further information on Nature Portfolio policies, see our Editorial Policies and the Editorial Policy Checklist.

Statistics

For all statistical analyses, confirm that the following items are present in the figure legend, table legend, main text, or Methods section.

n/a	Confirmed	
☐	☒	The exact sample size (*n*) for each experimental group/condition, given as a discrete number and unit of measurement
☐	☒	A statement on whether measurements were taken from distinct samples or whether the same sample was measured repeatedly
☐	☒	The statistical test(s) used AND whether they are one- or two-sided<br>*Only common tests should be described solely by name; describe more complex techniques in the Methods section.*
☒	☐	A description of all covariates tested
☐	☒	A description of any assumptions or corrections, such as tests of normality and adjustment for multiple comparisons
☐	☒	A full description of the statistical parameters including central tendency (e.g. means) or other basic estimates (e.g. regression coefficient) AND variation (e.g. standard deviation) or associated estimates of uncertainty (e.g. confidence intervals)
☐	☒	For null hypothesis testing, the test statistic (e.g. *F*, *t*, *r*) with confidence intervals, effect sizes, degrees of freedom and *P* value noted<br>*Give P values as exact values whenever suitable.*
☒	☐	For Bayesian analysis, information on the choice of priors and Markov chain Monte Carlo settings
☒	☐	For hierarchical and complex designs, identification of the appropriate level for tests and full reporting of outcomes
☒	☐	Estimates of effect sizes (e.g. Cohen's *d*, Pearson's *r*), indicating how they were calculated

Our web collection on statistics for biologists contains articles on many of the points above.

Software and code

Policy information about availability of computer code

Data collection	Igor Pro 8 (WaveMetrics): Patch-clamp recordings; Leica LAS-X (Core_3.7.4_23463): Confocal imaging; Zen 2.3 (black edition, v.14.0.9.201): Super resolution; Image Studio Lite software (version 5.2): Western blots.
Data analysis	Igor Pro 8 (WaveMetrics): Patch-clamp recordings; FIJI-ImageJ (NIH): Confocal images; ZEN 2.3 (blue edition, v.2.3.69.1000): Super resolution.

For manuscripts utilizing custom algorithms or software that are central to the research but not yet described in published literature, software must be made available to editors and reviewers. We strongly encourage code deposition in a community repository (e.g. GitHub). See the Nature Portfolio guidelines for submitting code & software for further information.

Data

Policy information about availability of data

All manuscripts must include a data availability statement. This statement should provide the following information, where applicable:

- Accession codes, unique identifiers, or web links for publicly available datasets
- A description of any restrictions on data availability
- For clinical datasets or third party data, please ensure that the statement adheres to our policy

Publicly available NIH database (GEO repository): Accession # GSE129241 [https://www-ncbi-nlm-nih-gov.ezproxy.u-pec.fr/geo/query/acc.cgi?acc=GSE129241]

Field-specific reporting

Please select the one below that is the best fit for your research. If you are not sure, read the appropriate sections before making your selection.

☒ Life sciences ☐ Behavioural & social sciences ☐ Ecological, evolutionary & environmental sciences

For a reference copy of the document with all sections, see nature.com/documents/nr-reporting-summary-flat.pdf

Life sciences study design

All studies must disclose on these points even when the disclosure is negative.

Sample size	At least >= 3 biological replicates were used. Sample sizes were chosen so that Standard Error (i.e. standard-deviation / square-root of number of samples) ≈< 1/10th of the Mean. This ensured large enough datasets from all experimental conditions to cover for biological variabilities.
Data exclusions	No data was excluded.
Replication	Multiple samples from at least >= 3 experimental batches were analyzed to ensure reproducibility. All attempts at replication were successful.
Randomization	Sample allocation was random.
Blinding	Except Figs. 3 and 5, investigators were not blinded to allocation during experiments or outcome assessments, because many assays required prior knowledge of drug identity during acute applications (Figs. 1, 2, 6, and 7), transgene combinations (Figs. 1 and 7), or sample collections and processing at specific time-intervals (Fig. 4). All phenotypes were robust and highly reproducible in multiple independent experimental batches. In addition, our assays took stringent quantitative measures with appropriate statistics that minimized the chances for observer bias.

Reporting for specific materials, systems and methods

We require information from authors about some types of materials, experimental systems and methods used in many studies. Here, indicate whether each material, system or method listed is relevant to your study. If you are not sure if a list item applies to your research, read the appropriate section before selecting a response.

Materials & experimental systems

n/a	Involved in the study	
☐	☒	Antibodies
☐	☒	Eukaryotic cell lines
☒	☐	Palaeontology and archaeology
☐	☒	Animals and other organisms
☒	☐	Human research participants
☒	☐	Clinical data
☒	☐	Dual use research of concern

Methods

n/a	Involved in the study	
☒	☐	ChIP-seq
☒	☐	Flow cytometry
☒	☐	MRI-based neuroimaging

Antibodies

Antibodies used

1. PRIMARY ANTIBODIES:
Calretinin, Species: Goat, Vendor: Swant, Catalog # CG1 (Lot # 1§.1), Dilution: 1:1000
EGFP, Species: Chicken, Vendor: Aves Labs, Catalog #GFP-1020, Dilution: 1:1000
GABRA3, Species: Rabbit, Vendor: Abclonal, Catalog #A11636, Dilution: 1:500
GAD65, Species: Rabbit, Vendor: Abclonal, Catalog #A0971, Dilution: 1:500
GAD67, Species: Rabbit, Vendor: Abclonal, Catalog #A2938, Dilution: 1:500
Gephyrin [#1], Species: Mouse, Vendor: Synaptic Systems, Catalog #147011 (Clone: mAb7a), Dilution: 1:500
Gephyrin [#2], Species: Mouse, Vendor: Synaptic Systems, Catalog #147111 (Clone: 3B11), Dilution: 1:500
Homer-1 [#1], Species: Mouse, Vendor: Synaptic Systems, Catalog #160011 (Clone: 2G8), Dilution: 1:500
Homer-1 [#2], Species: Rabbit, Vendor: Synaptic Systems, Catalog #160002, Dilution: 1:500
HuNu, Species: Mouse, Vendor: Millipore Sigma, Catalog #MAB1281 (Clone: 235-1), Dilution: 1:500
MAP2, Species: Chicken, Vendor: Abcam, Catalog #Ab5392, Dilution: 1:1000
Neuroligin-2, Species: Rabbit, Vendor: Synaptic Systems, Catalog #129203, Dilution: 1:500
RFP, Species: Rabbit, Vendor: Rockland, Catalog #600-401-379, Dilution: 1:500
Synapsin-1 [#1], Species: Mouse, Vendor: Synaptic Systems, Catalog #106011 (Clone: 46.1), Dilution: 1:1000
Synapsin-1/2 [#2], Species: Guinea Pig, Vendor: Synaptic Systems, Catalog #106004, Dilution: 1:500
Synapsin-1/2 [#3], Species: Rabbit, Vendor: Synaptic Systems, Catalog #106002, Dilution: 1:500
Tuj1 (βIII-tubulin), Species: Mouse, Vendor: BioLegend, Catalog #801202 (Clone: TUJ1), Dilution: 1:400/1000
vGLUT1, Species: Rabbit, Vendor: Synaptic Systems, Catalog #135303, Dilution: 1:500
vGAT [#1], Species: Mouse, Vendor: Synaptic Systems, Catalog #131011 (Clone: 117G4), Dilution: 1:500
vGAT [#2], Species: Rabbit, Vendor: Synaptic Systems, Catalog #131003, Dilution: 1:500

2. SECONDARY ANTIBODIES:
Alexa Fluor 568 donkey anti-goat (Invitrogen, Catalog #A11057) Dilution: 1:250
Alexa Fluor 488 goat anti-mouse (Invitrogen, Catalog #A11029) Dilution: 1:250-2000
Alexa Fluor 546 goat anti-mouse (Invitrogen, Catalog #A11030) Dilution: 1:1000-2000
Alexa Fluor 647 goat anti-mouse (Invitrogen, Catalog #A32728) Dilution: 1:1000-2000
Alexa Fluor 488 goat anti-rabbit (Invitrogen, Catalog #A11034) Dilution: 1:1000-2000
Alexa Fluor 488 donkey anti-rabbit (Invitrogen, Catalog #A21206) Dilution: 1:250
Alexa Fluor 546 goat anti-rabbit (Invitrogen, Catalog #A11035) Dilution: 1:1000-2000
Alexa Fluor 594 goat anti-rabbit (Invitrogen, Catalog #A11037) Dilution: 1:250
Alexa Fluor 647 donkey anti-rabbit (Invitrogen, Catalog #A31573) Dilution: 1:1000-2000
Alexa Fluor 488 goat anti-chicken (Invitrogen, Catalog #A11039) Dilution: 1:1000-2000
Alexa Fluor 546 goat anti-chicken (Invitrogen, Catalog #A11040) Dilution: 1:1000-2000
Alexa Fluor 647 goat anti-chicken (Invitrogen, Catalog #A21449) Dilution: 1:1000-2000
Alexa Fluor 488 goat anti-guinea pig (Invitrogen, Catalog #A11073) Dilution: 1:1000-2000
Alexa Fluor 555 goat anti-guinea pig (Invitrogen, Catalog #A21435) Dilution: 1:1000-2000
Alexa Fluor 647 goat anti-guinea pig (Invitrogen, Catalog #A21450) Dilution: 1:1000-2000

Validation

All antibodies for this study were validated by their vendors using immunostainings and/or western blots, and sometimes also using cells or tissues from animal knockouts for respective proteins (please see vendor websites with catalog # for validation statements). In addition, we also independently verified the specificities of several antibodies in our experimental system (Supplementary Fig. S3).

Eukaryotic cell lines

Policy information about cell lines

Cell line source(s)

HEK 293T cells (Takara Bio USA);
Human ES cells (H1-line, WiCell WA01);
iPS cells (WTC-11 line, generously gifted by Dr. Michael E. Ward, NIH; Publication: Wang et al., 2017, PMID: 28966121).

Authentication

Commercially available H1-ES cells and HEK 293T cells were not authenticated;
WTC-11 iPS cells were validated by neural differentiation upon doxycycline induction.

Mycoplasma contamination

H1-ES cells tested negative for mycoplasma contamination; WTC-11 iPS cell line was not tested for mycoplasma.

Commonly misidentified lines
(See ICLAC register)

No commonly misidentified cell lines were used in the study.

Animals and other organisms

Policy information about studies involving animals; ARRIVE guidelines recommended for reporting animal research

Laboratory animals

Mouse, JAX strain CBA/CaJ, Male and Female, Postnatal day P2-P32;
Maintained at 12 hours dark / 12 hours light, ambient temperature between 21°C to 24°C, and humidity between 30% to 60%.

Wild animals

No wild animals were used in the study.

Field-collected samples

No field-collected samples were used in the study.

Ethics oversight

Experiments in mice were approved by the Institutional Animal Care and Use Committee (IACUC) of University at Buffalo.

Note that full information on the approval of the study protocol must also be provided in the manuscript.
